# Dynamic Characteristics and Anti-slip Grasping of Two-Finger Translational Manipulator

**DOI:** 10.3389/fnbot.2021.684317

**Published:** 2021-06-10

**Authors:** Wei Liu, Jin Cheng, Ping Wan, Cheng Jing, Yongheng Ma, Keshiting Chen

**Affiliations:** School of Automotive Engineering, Yancheng Institute of Technology, Yancheng, China

**Keywords:** industrial robot, kinematic analysis, dynamic characteristics, 3D modeling, Robot Studio

## Abstract

Aiming at the problem of deformation and slip of disc-shaped rubber gasket in the process of grasping, a two-finger translational manipulator based on ABB1410 robot is designed. The kinematics model of the two-finger translational manipulator is established, and the geometric relationship between the motor angle and grasp position is obtained. Based on the two-dimensional force sensor, the dynamic characteristics of the two-finger translational manipulator were studied, and the relationship between the grasping force and the deformation and slip of the disc-shaped rubber gasket was obtained. A prototype of two-finger translational manipulator is developed. The experimental results show that when the grasping force is 5 N, small deformation and stable grasping can be achieved. The grasping and handling process of disk-shaped rubber gasket is designed based on Robot Studio software, and the verification experiments were carried out. The experimental results show that the system can achieve the small deformation and stable grasping of flexible objects, which is consistent with the simulation results. The research can provide theoretical and experimental basis for the design of automation system structure and process control.

## Introduction

Under the background of Industry 4.0 era, intelligence has become the mainstream, and industrial robot has become the key to its transformation and upgrading, and its future development can be expected (Yin, 2020). The robot improves the safety of production, labor efficiency, quality stability, and reduces the material consumption rate and human resource cost investment (Ma, 2020). The Robot needs a variety of end-effectors to grasp materials. In order to make the manipulator structure complete the task of stably grasping the stored parts, scholars at home and abroad have carried out a lot of research on the main body, drive system, and control system of industrial robots, and have achieved relevant results (Duan et al., 2019).

Application of arc welding robot and yaskawa universal industrial robot built experimental platform, the collaboration of two systems on the relations between the two robots in master-slave motion is studied, this paper proposes a model based on variable impedance of internal and external position/force hybrid control strategy, and successfully developed for no fixture welding robot teaching method (Xiao et al., 2020). And they established the dynamic modeling and simulation of the manipulator, realized the real-time control of the manipulator, and had higher efficiency and accuracy (Su and Kong, 2008). Designed a five degree of freedom mechanical gripper. The structure of the gripper was designed, the clamping force of the gripper was calculated and analyzed. The designed gripper can achieve stable grasping effect (Jiao, 2019). Slightly improved the ordinary two-finger gripper by adding a spring element, which changed the hard contact into soft contact and improved the grasping performance (Hu et al., 2014). Adopted a hydraulically driven end-effector in mechanized watermelon picking to avoid damage caused by unstable clamping force, accurately control the clamping force, and achieve the purpose of stable grabbing (Zhang et al., 2020). Designed an adaptive gripper based on the under-actuated system, which simplified the complex structure of the manipulator, and was able to complete the grasping of the envelope of cylindrical and square-shaped objects and pinching of relatively fine square-shaped objects (Lei and Liu, 2020). They designed a spring passive adaptive finger mechanism, established the dynamic model and carried out the kinematics simulation, which provided an effective basis for the structural design of the manipulator (Hamidreza et al., 2018). Design a three-link finger for the fabrication of a two-fingered gripper, which can complete most grasping tasks and grasp various objects of unknown nature. It is helpful to the structural design of this paper (Lionel, 2019). He based on the traditional translation gripper, changed the structure of the manipulator, and designed an adaptive under-actuated manipulator. The experimental results show that the under-actuated mechanism can adapt to different objects and different complex surfaces when grasping.

At the same time, scholars at home and abroad have carried out in-depth research on the sliding problem of the manipulator (Varun et al., 2021). Used a control structure composed of safety sensors and related systems is used to complete the assembly task on a continuously moving production line (Yi et al., 2021). With the increase of the contact force, the deformation of the fingertip increases. Through the detection of the force sensor, the impact of the contact force is avoided to increase and the safety clamping is ensured (Shuangji et al., 2018). Designed a robot claw control system based on closed-loop position and force feedback. The angular displacement sensor and control algorithm were used to achieve stable grasping (Ma et al., 2008). Used neural network inverse system method to solve the problem of dynamic characteristics of flat ring type two-dimensional force sensor and eliminate the serious coupling phenomenon between dimensions. The composite measurement system composed of dynamic compensator designed by this method can eliminate the coupling phenomenon between dimensions and greatly improve the dynamic characteristics of the system (He et al., 2014). Developed a two-dimensional force sensor array test system using cantilever force sensor, and verified the reliability of the sensor and the system (Tang and Luan, 2019). Judged the change of grasping force through the output signal of PVDF piezoelectric film sensor, and finally realized the fruit grasping through the signal feedback of the sensor (Han et al., 2019). Designed a six-dimensional force sensor with high sensitivity and large range, and measured the sensor in the experiment. The results showed that the maximum range of the force channel of the sensor was 3,000 N, the measurement sensitivity was >0.83 mV/V, and the coupling error between the residual dimensions of the decoupled sensor was <1.2%, which could be well-used in assembly experiments (Li et al., 2011). Added a new type of slip detection sensor and adopted a layered control strategy to reduce the impact force during grasping and realize the autonomous grasping function (Sun et al., 2020). Designed a two-dimensional force sensor based on fiber Bragg grating, studied the sensitivity, linearity, coupling error between dimensions and repeatability error of the sensor, and successfully reduced the error, carried out high-precision force control feedback, and realized stable grasping (Song et al., 2018). Introduced a slip sensor into the end-effector in order to make the gripper have stronger adaptability and realize the non-slip and stable grasping of the object. The results show that high sensitivity and linearity are used to detect the relative slip between the object and the gripper.

Based on the body of ABB1410 robot, the end-effector structure of grasping flexible workpiece is designed, and the stable grasping problem under small deformation is studied. The rest of this paper is arranged as follows: the second chapter design the structure of the two-finger translational manipulator, the third chapter studies the dynamic characteristics of the two-finger translational a manipulator, the fourth chapter carries on the stable grasping experiment, and finally the conclusion is drawn in the fifth chapter.

## Design the Structure of Two-Finger Translational Manipulator

### Structural Design, 3D Modeling, and Assembly of Two-Finger Translational Manipulator

In this paper, based on ABB1410 ontology, the grasping object is a disk-shaped rubber gasket, and the stable grasping of the flexible workpiece is required under the premise of small deformation. In order to meet the functional requirements of the clamping corresponding objects, and to avoid the complicated motion control caused by the complex structure, the gripper is designed as a two-finger translational manipulator claw. The device is mainly composed of two parts: power transmission mechanism and grasping device. The transmission mechanism is composed of a fixed seat, a connecting rod and a connecting piece. The grasping device consists of a slider and a gripper. The size and structure of each part of the transmission mechanism and grasping device are shown in Table 1.

**Table 1:** Dimension structure table of important components of two-finger translational manipulator.

**Component**	**Size structure**
Fixed seat	Semi-circular fixed seat with thickness of 6 mm and radius of 50 mm
Connecting rod	34 mm long and 2 mm thick connecting rod
Connector	Semi-circular arc connector with a center distance of 27 mm and a thick 2 mm
Slider	T-type slider with upper bottom of 20 mm, lower bottom of 10 mm, height of 35 mm and thickness of 3 mm
Gripper	Gripper with slot for pressure sensor

**Figure 1:**
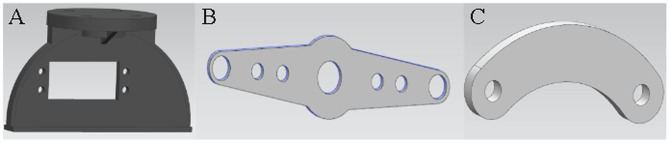
**(A)** 3D modeling of fixed seat **(B)** 3D modeling of connecting rod **(C)** 3D modeling of connector.

**Figure 2:**
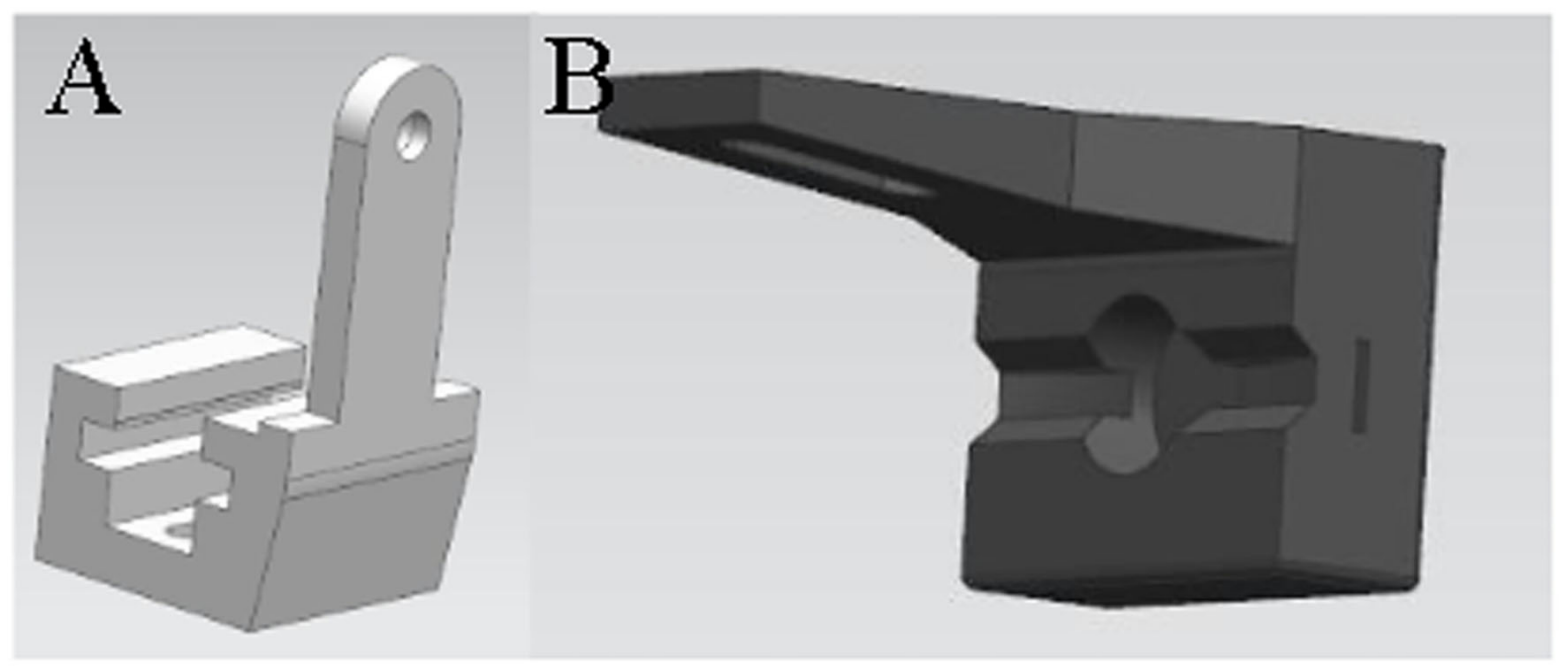
**(A)** 3D modeling of slider **(B)** 3D modeling of gripper.

According to the dimensions of the above parts, 3D modeling is carried out by UG software. The 3D drawings of the parts of the transmission mechanism are shown in Figure 1, and the 3D drawings of the parts of the grasping device are shown in Figure 2.

Finger type translational manipulator design in this paper is in the mechanical transmission parts of rotary motion is converted into the linear motion of the object, the course of the finger type translational movement of manipulator, is driven by a motor axis of rotation, the intermediate connecting rod drive on both sides of the pit, respectively, from moving up and down, to drag along the linear bearing mechanical wrist part near each other, this process is the clamping action of the manipulator, otherwise, it realizes the loosening action. The whole connecting rod is connected by special bearings to reduce friction between each other.

**Figure 3:**
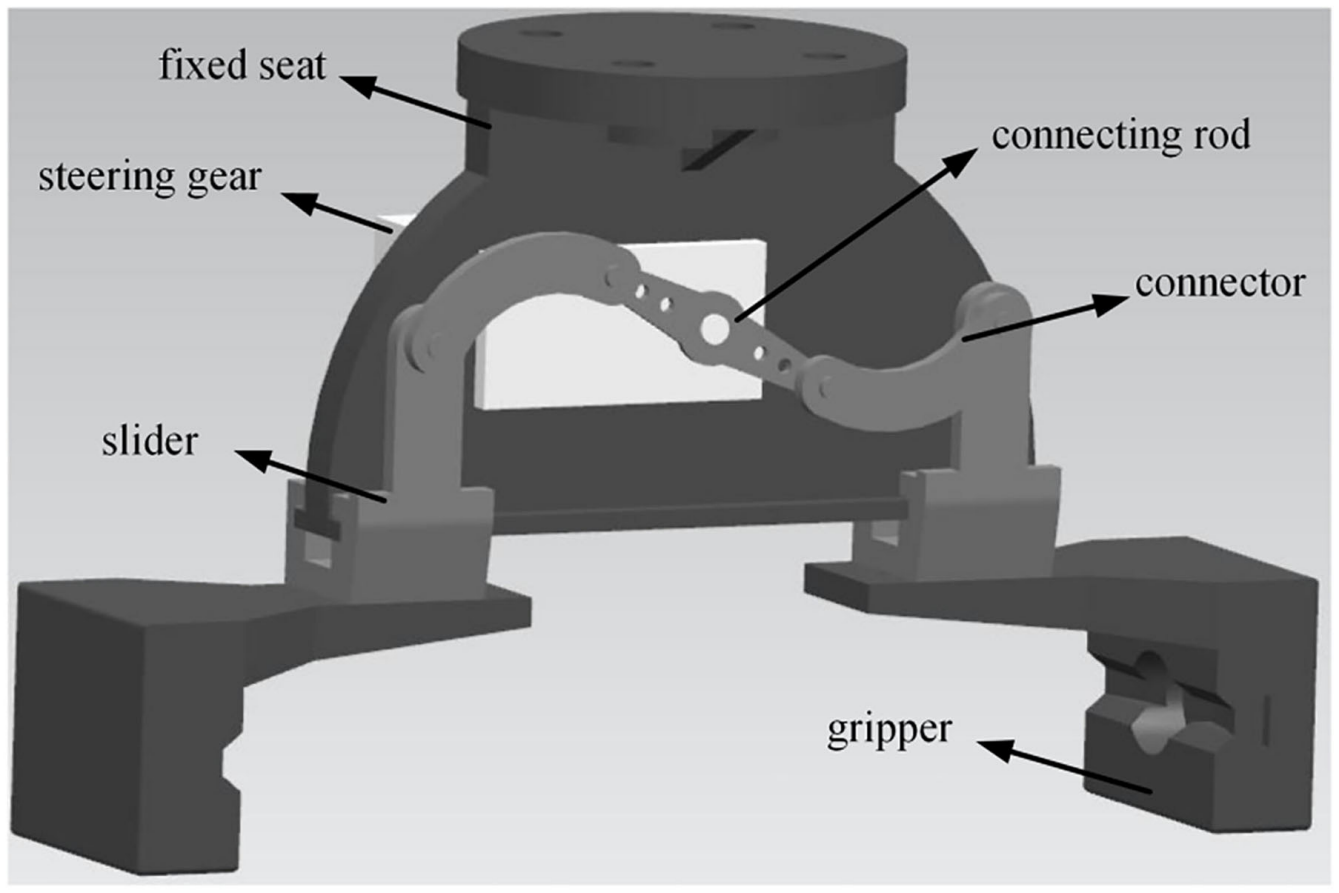
3D assembly of two-finger translational manipulator.

The parts drawing is assembled into the finished assembly drawing through UG 3D software, and the final assembly effect drawing is shown in Figure 3.

### Kinematic Analysis

**Figure 4:**
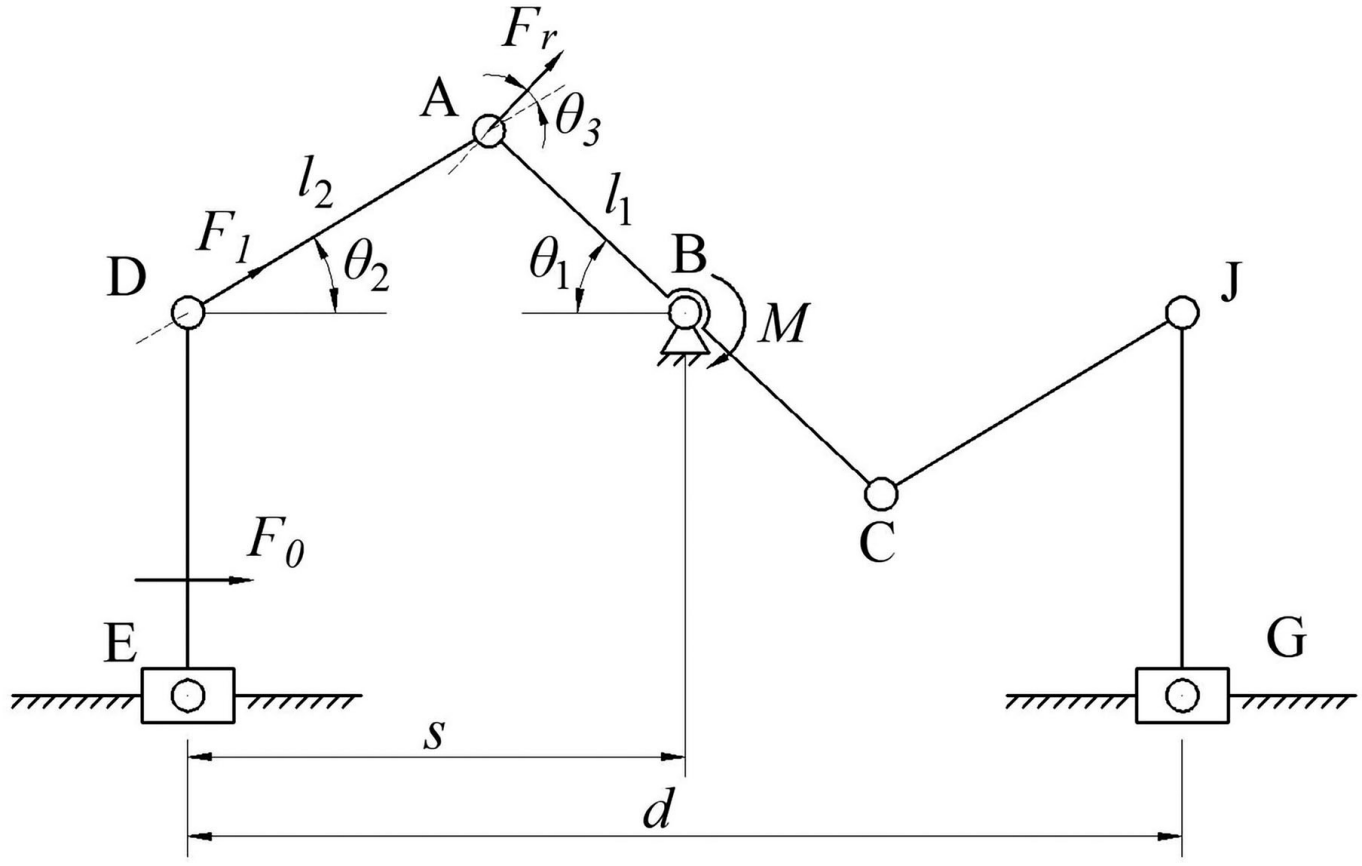
Structure diagram of two-finger translational manipulator.

In order to solve the relationship between input and output in the grasping device, the kinematics of the two-finger translational manipulator was analyzed. The driving motor is fixed with the guide rail, the connecting piece is a connecting rod, and the hand claw is simplified to a plane guide rail slider mechanism. The structure diagram is shown in Figure 4.

Because the structure of the end effector in this paper is centrosymmetric, it is enough to analyze half of the structure diagram in the process of kinematic analysis. In Figure 1, AB = *l*_1_, AD = *l*_2_, point D, point B, and point J are on the same horizontal line, the angle between AB and horizontal direction is θ_1_, and the angle between AD and horizontal direction is θ_2_. B is the rocker arm of the steering gear, which is used as the prime mover, the nominal torque is M, The force at A perpendicular to the direction AB of the rod is *F*_*r*_, The angle between the force *F*_*r*_ and the force *F*_1_ in the AD direction of the connecting rod is θ_3_, and the force shared by the slider E in the horizontal direction is *F*_0_. Rod AC moves counterclockwise around point B, The angle between the rod AC and the horizontal direction is in the range of 0–180 degrees. Connecting rod AD moves with the rotation of rod AC, connecting rod AD moves with rod DE in horizontal direction, the distance of BE in vertical direction is *h*, the distance of BE in horizontal direction is *s*, the distance of EG in horizontal direction is *d*.

According to Figure 1, three points A, B, and D can form a whole triangle structure, which decomposes *l*_1_ and *l*_2_ horizontally, the formula is:

(1) $$\begin{array}{l} s=l_{1}\cos\theta_{1}+l_{2}\cos\theta_{2} \end{array}$$

The distance between the sliders is *d* = 2*s* so:

(2) $$\begin{array}{l} d=2l_{1}\cos\theta_{1}+2l_{2}\cos\theta_{2} \end{array}$$

In Figure 1, *M* is torque, and torque is the product of force and moment arm, so:

(3) $$\begin{array}{l} M=F_{r}\cdot l_{1} \end{array}$$

In formula (3), the force shared by *F*_*r*_ in the direction of AD of the connecting rod is:

(4) $$\begin{array}{l} F_{1}=F_{r}\cdot \cos\theta_{3} \end{array}$$

In formula (4), the force *F*_1_ is decomposed in the horizontal direction, and the formula is:

(5) $$\begin{array}{l} F_{0}=F_{1}\cdot \cos\theta_{2} \end{array}$$

In the three-dimensional assembly drawing of the two-finger translational manipulator, the rotational motion part of the machine part and the linear motion part of the machine part are arranged symmetrically. According to the structure diagram of the claw, the displacement range of half the gripper can be deduced, thus the grasping range of the whole gripper. Formula (2) is the distance between two sliders. When θ_1_ takes 0°, the rod AC is on the horizontal line, and the points D, A, and B are on the same horizontal line, and *l* reaches the maximum. When θ_1_ takes 110°, *l* reaches the minimum. The range of motion between the two sliders is 33–86 mm. There is a distance of 35 mm between the slider and the gripper, so the grasping range of the whole end effector is 103–156 mm, the geometric relationship between motor angle and grasp position of two-finger translational manipulator is shown in Table 2.

**Table 2:** Geometric relationship between motor angle and grasp position of two-finger translational manipulator.

	**Experiment 1**	**Experiment 2**	**Experiment 3**	**Experiment 4**	**Experiment 5**
Motor angle	0°	20°	50°	80°	110°
Grasp position	156 mm	150 mm	138 mm	120 mm	103 mm

## The Dynamic Characteristics of the Two-Finger Translational a Manipulator

**Figure 5:**
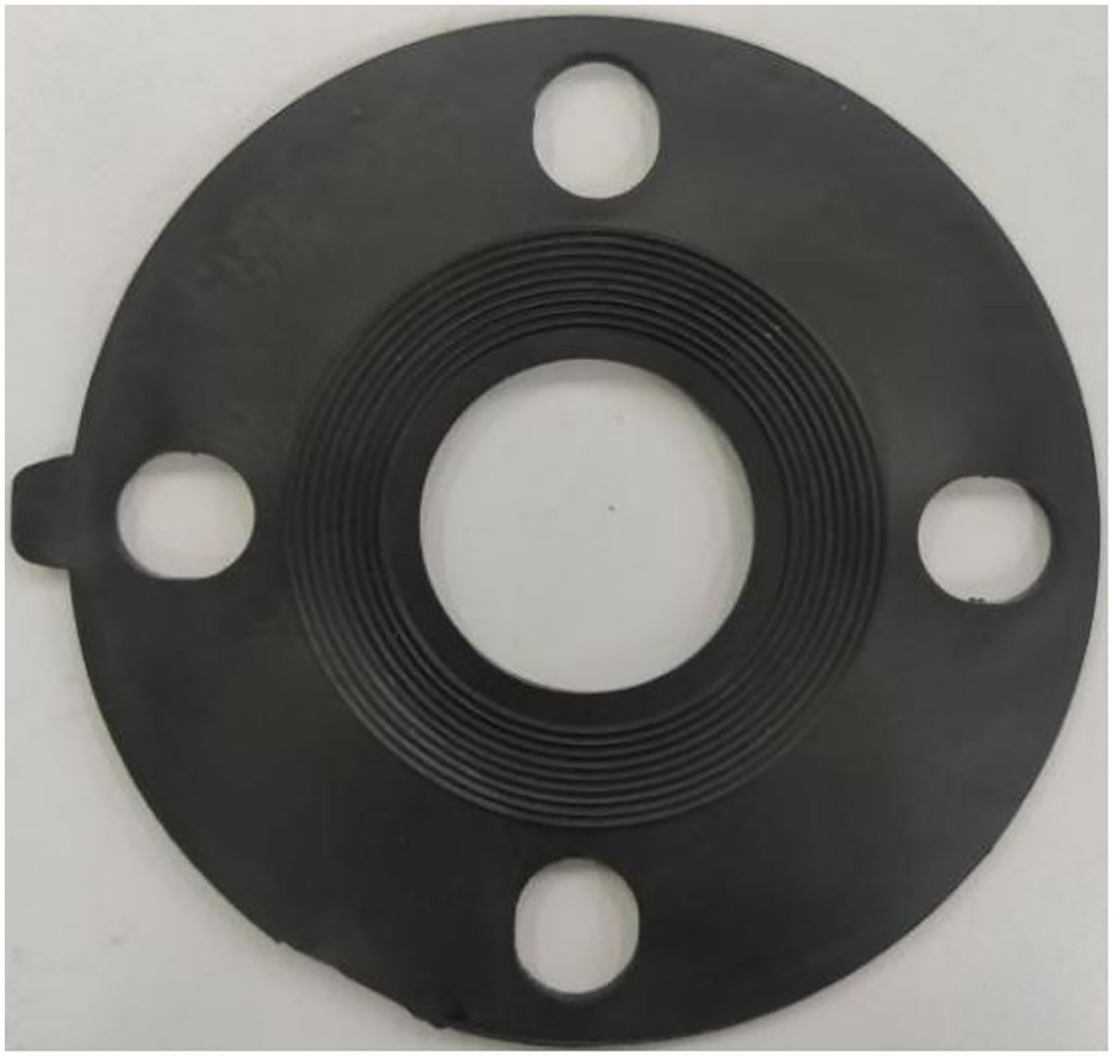
Physical drawing of rubber gasket.

The object of this experiment is a disk-shaped rubber gasket, with a diameter of 128 mm, as shown in Figure 5.

In the whole process of grasping, how to grasp stably and prevent the deformation of the object is a difficult problem. In order to solve this problem and achieve the purpose of anti-slip and anti-deformation, a resistive two-dimensional force sensor is added to the end effector, the clamping force and tangential force information can be sensed in the process of grasping, and the clamping force can be adjusted dynamically in real time. The test system consists of software and hardware. The software has the functions of data acquisition and storage. The hardware part is composed of two-finger translational manipulator, steering gear, two-dimensional force sensor, single-chip microcomputer STM32F407ZG, etc. When the measuring object is in contact with the sensor, the voltage signal induced by the sensor is sorted out by the signal conditioning circuit and then entered into the STM32F407ZG data acquisition module for A/D conversion, and then the data is sorted out and displayed by it. The selected resistance sensor has the advantages of fast response, easy adjustment and change of shape and sensitivity, simple acquisition and convenient installation.

**Figure 6:**
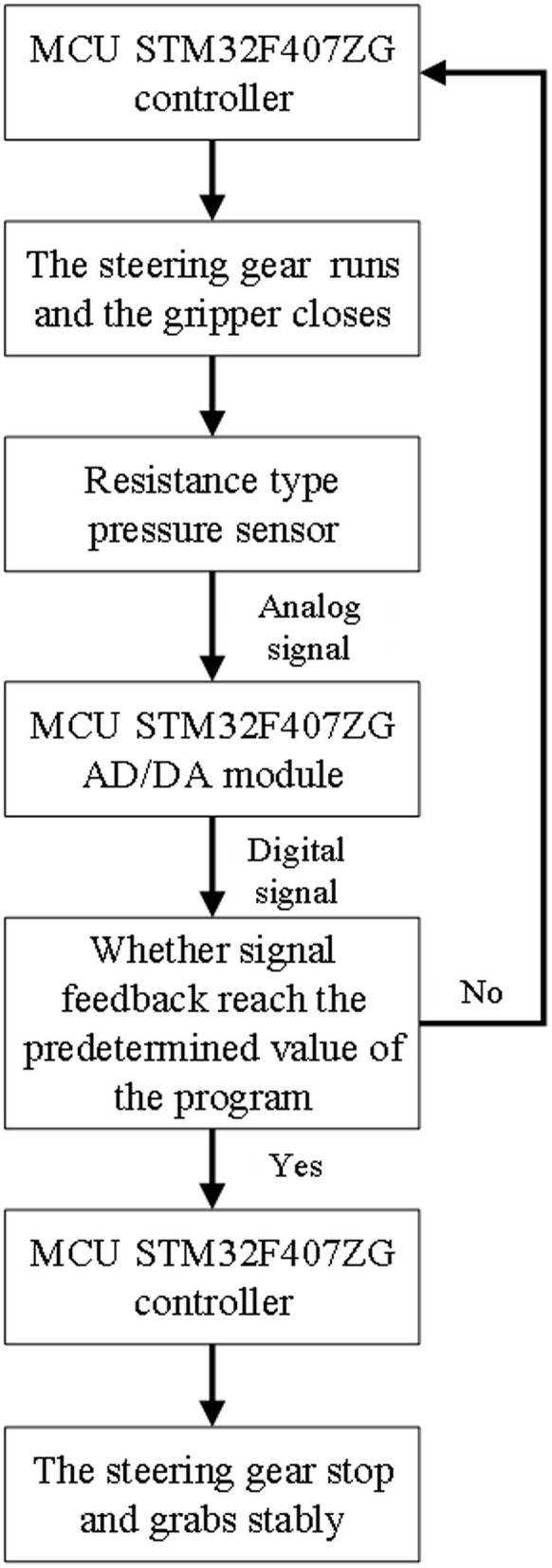
Schematic diagram of the control system.

In order to complete the grasping task and determine the size of grasping force to achieve the effect of no deformation of grasping parts, many experiments need to be carried out. First of all, the program compiled successfully is burned to the microcontroller STM32F407ZG of the experimental equipment through the software STM32CubeIDE. Press the up button to open the end-effector to the maximum, and then press the reset button and press the down button to close the end-effector of the two-finger translational manipulator until the minimum grasp position. Secondly, the range of F0 is obtained according to formula (5), and the range of grasping force F0 is 0–10 N. At the same time, the part grasping experiment is carried out within the range of this force to further obtain the appropriate grasping force. Finally, the appropriate grasping force is selected to achieve the purpose of the experiment. Schematic diagram of the control system between the sensor and the SCM is shown in Figure 6.

**Figure 7:**
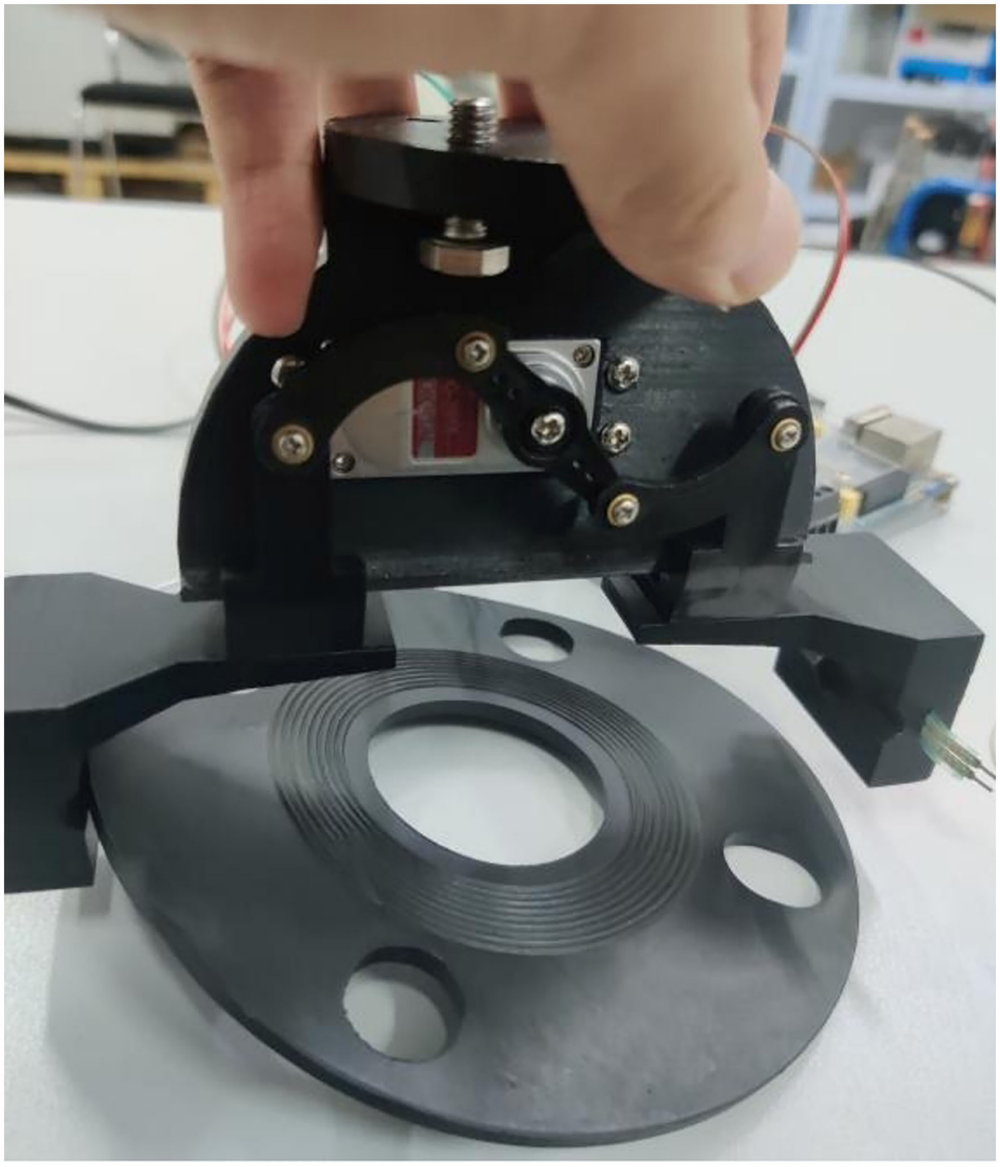
The slippage effect picture of the gasket during the grasping test.

**Figure 8:**
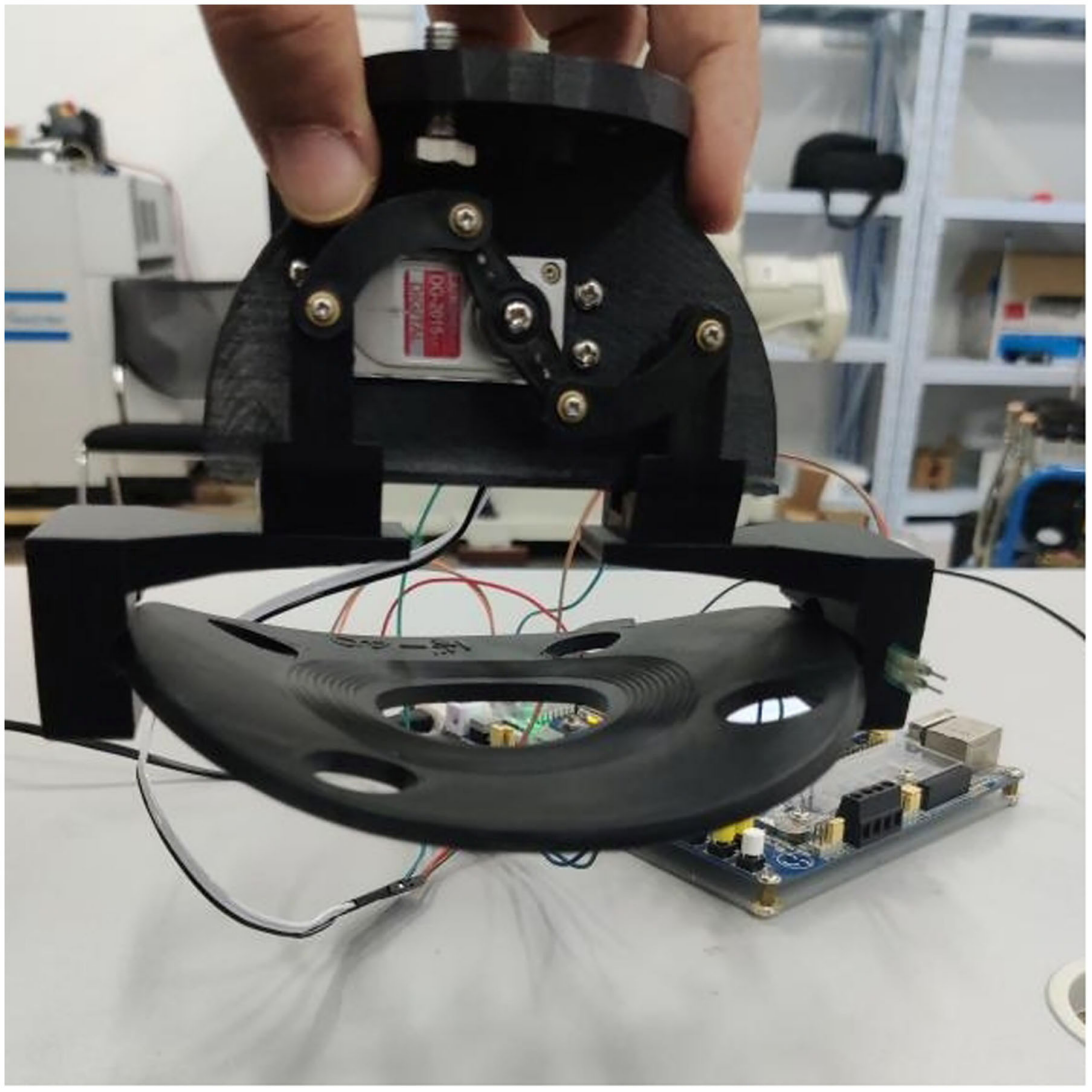
The gasket produces the deformation effect during the grasping test.

**Figure 9:**
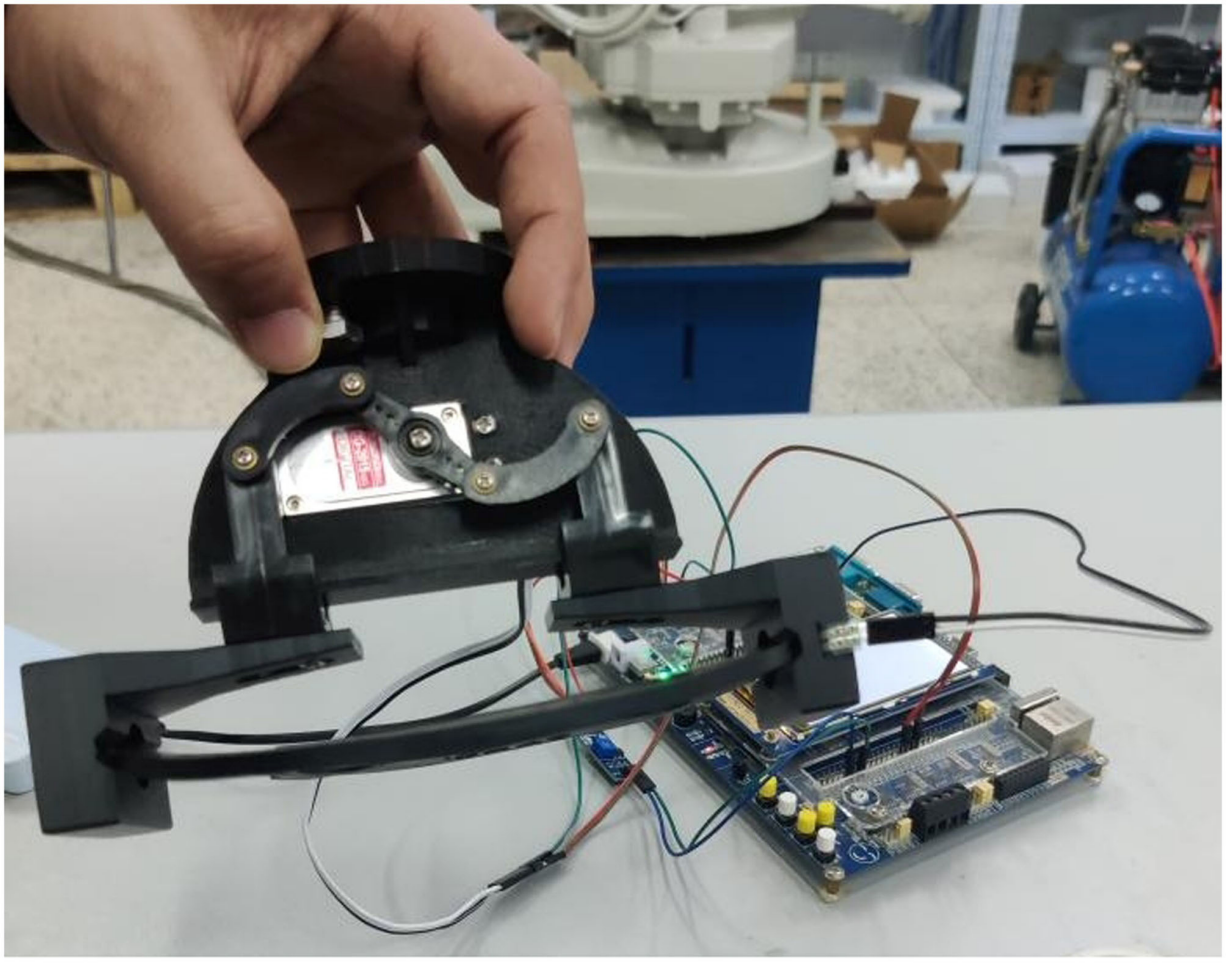
Effect of successfully grasping gasket.

Figure 7 Slippage occurs during grabbing and cannot be grasped stably. In Figure 8, there is no slip during grasping, but the deformation of the part is large. In Figures 7, 8, the controller directly drives the steering gear, slippage and part deformation occur in the process of grasping, and the grasping effect cannot be achieved. In this experiment, the groove of the sensor is designed on the inside of the clamping claw, during grasping, the sensor signal is transmitted to the controller through the AD/DA module of SCM STM32F407ZG by pressing the gasket, so as to control the rotation of the steering gear and finally realize stable grasping, as shown in Figure 9.

**Figure 10:**
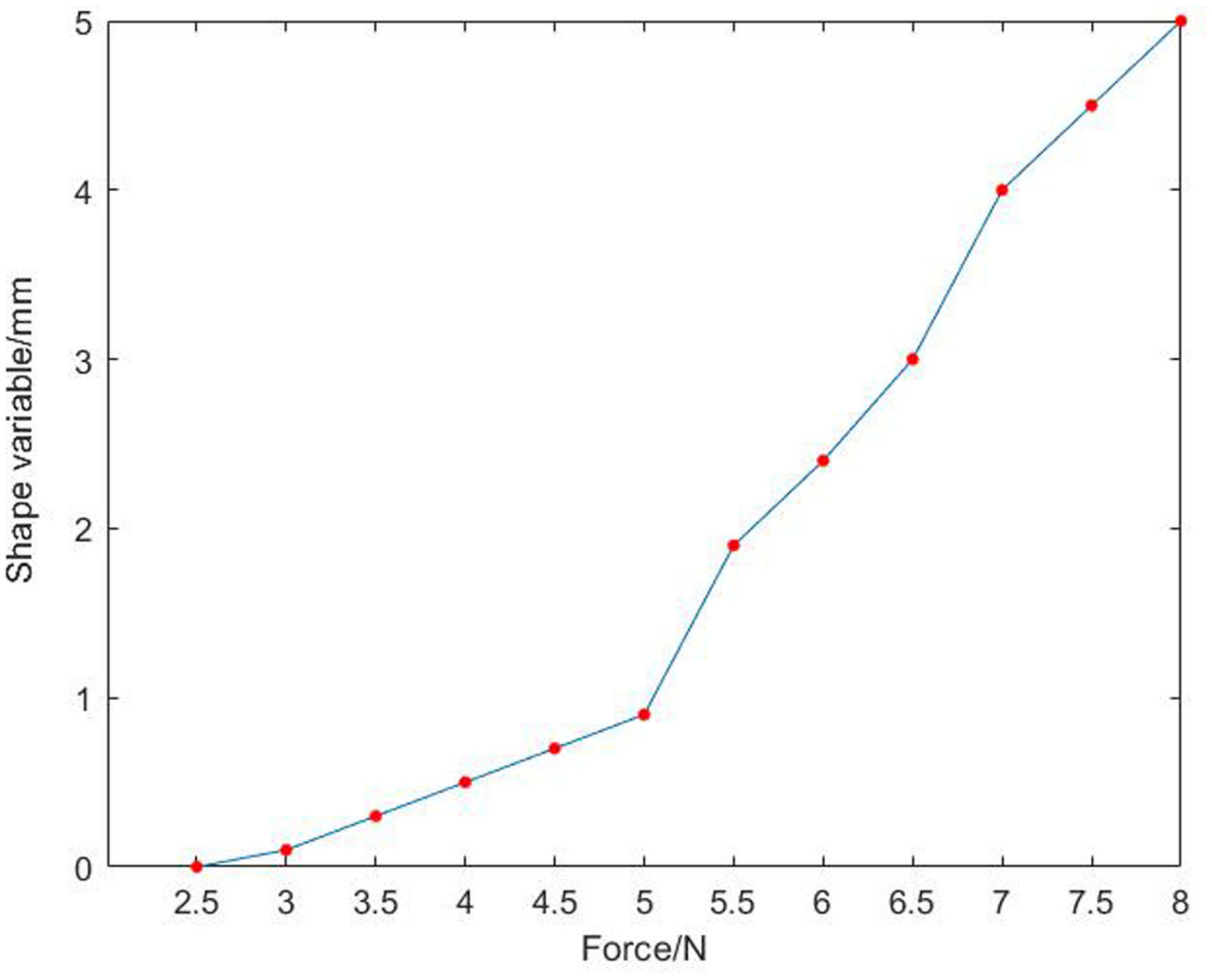
Diagram of the relationship between force and gasket deformation after the installation of sensor.

**Figure 11:**
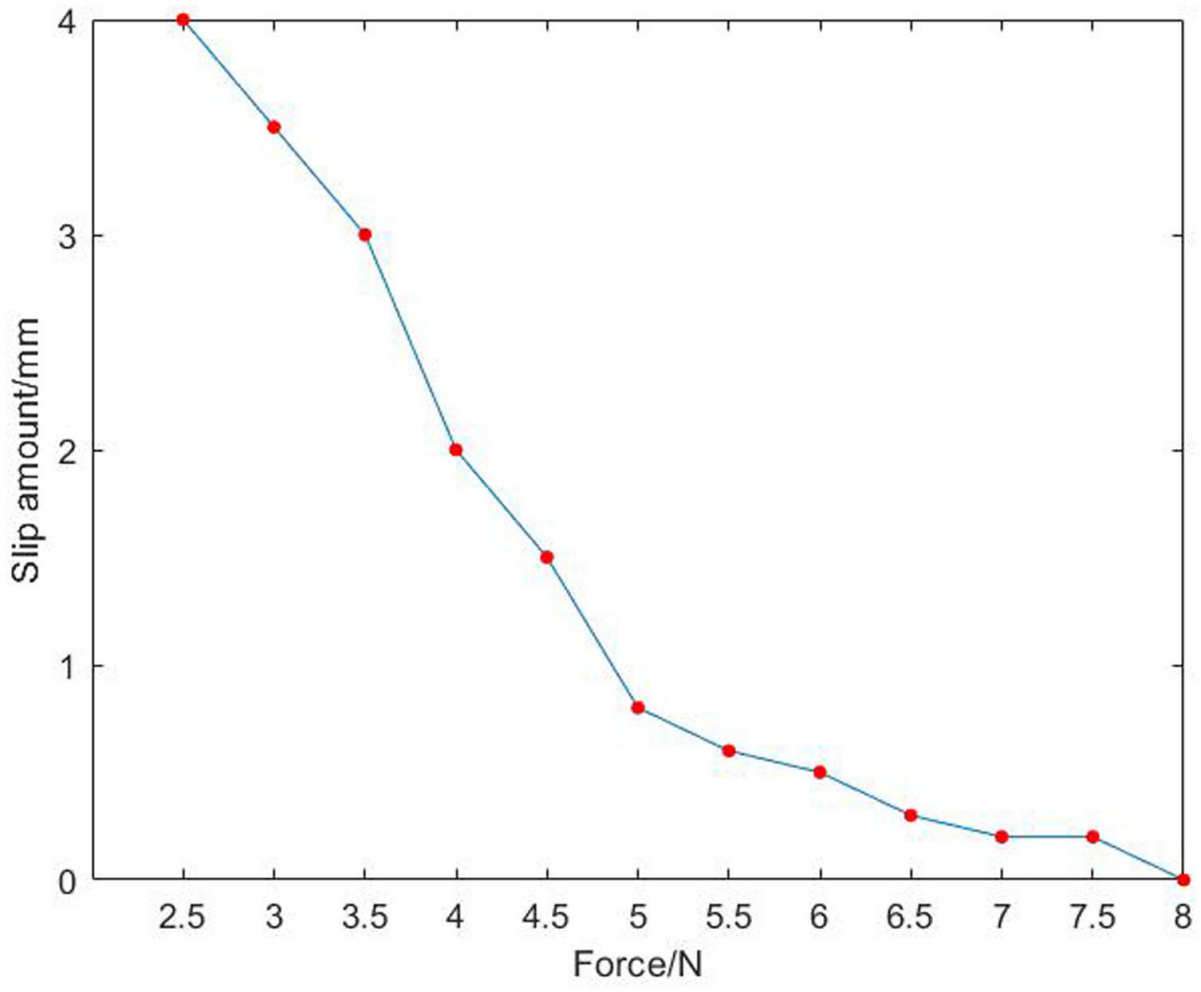
Diagram of the relationship between force and slip after the installation of a sensor.

In Figure 9, after installed a resistive two-dimensional force sensor, the parts can be grasped stably and the deformation is small. Through formula (5), and within the effective grasping force range, the fixed load is applied successively, and the data of multiple grasping force and part deformation and slip as well as the corresponding graph of the relationship are recorded simultaneously. The experimental data of grasping force, component shape variables and slip amounts are shown in Table 3. The corresponding relationship between force and shape variable is shown in Figure 10, and the corresponding relationship between force and slip amount is shown in Figure 11.

**Table 3:** Experimental data of grasping force, component shape variables and slip amount.

**Samples**	**1**	**2**	**3**	**4**	**5**	**6**	**7**	**8**	**9**	**10**	**11**	**12**
Grab force (N)	2.5	3.0	3.5	4.0	4.5	5.0	5.5	6.0	6.5	7.0	7.5	8.0
Shape variable (mm)	0	0.1	0.3	0.5	0.7	0.9	1.9	2.4	3.0	4.0	4.5	5.0
Slip amount (mm)	4.0	3.5	3.0	2.0	1.5	0.8	0.6	0.5	0.3	0.2	0.2	0

In Figure 10, the shape variable of the part increases with the increase of the grasping force. In Figure 11, the diagram is approximately linear, and the slip caused by grasping decreases with the increase of gripping force. Combined with the two figures, when the grasping force is 4.5 N, the shape variable of the part is small, but the slip is very large; when the grasping force is 5.5 N, the slip is small, but the shape variable of the part is large. Therefore, when the grasping force is 5 N, the shape variable and slip of the part meet the experimental requirements, and the final anti-skid effect can be achieved.

## Stable Grasping Simulation and Experiment

**Figure 12:**
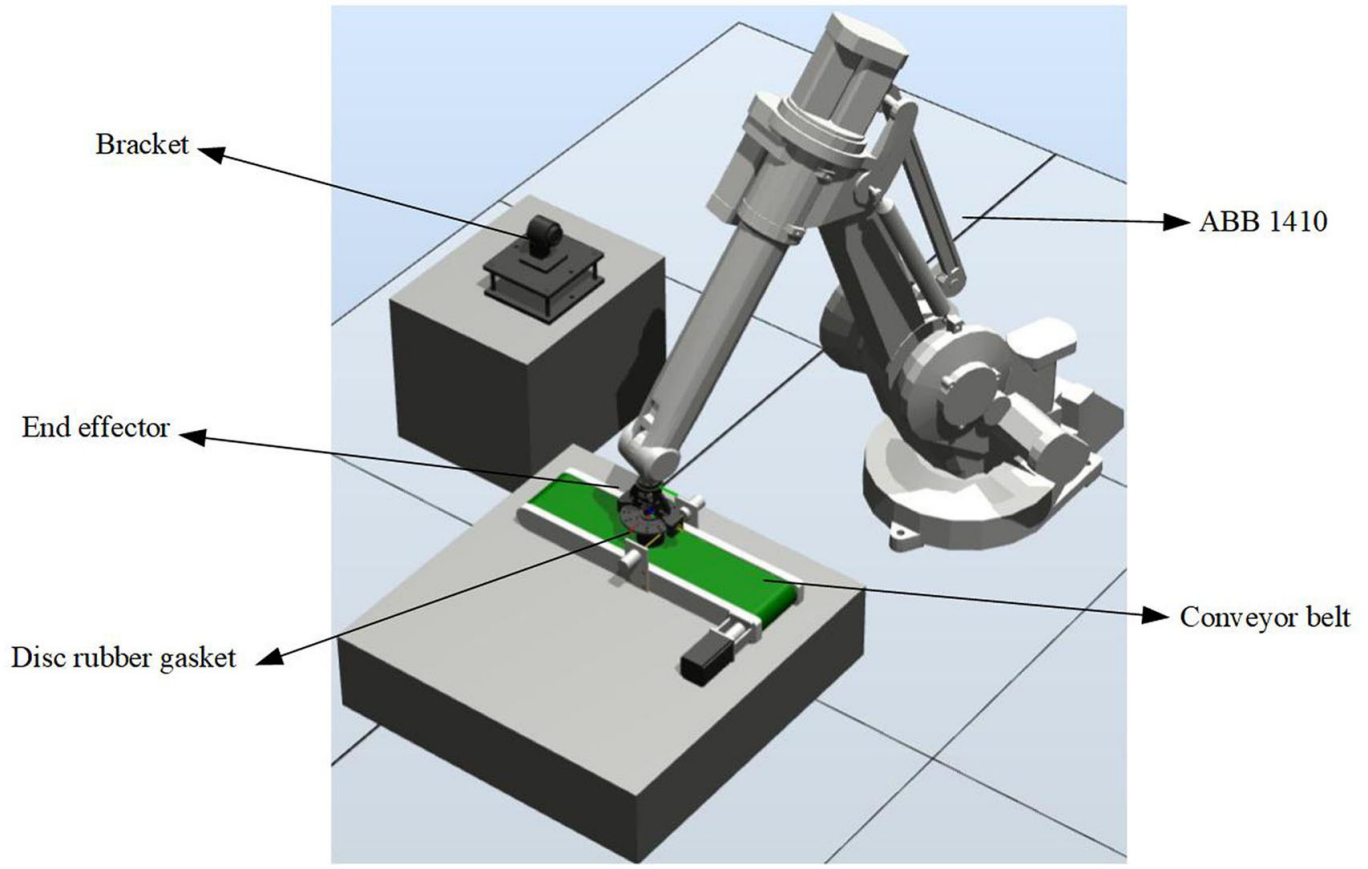
Robot Studio grasp work map.

**Figure 13:**
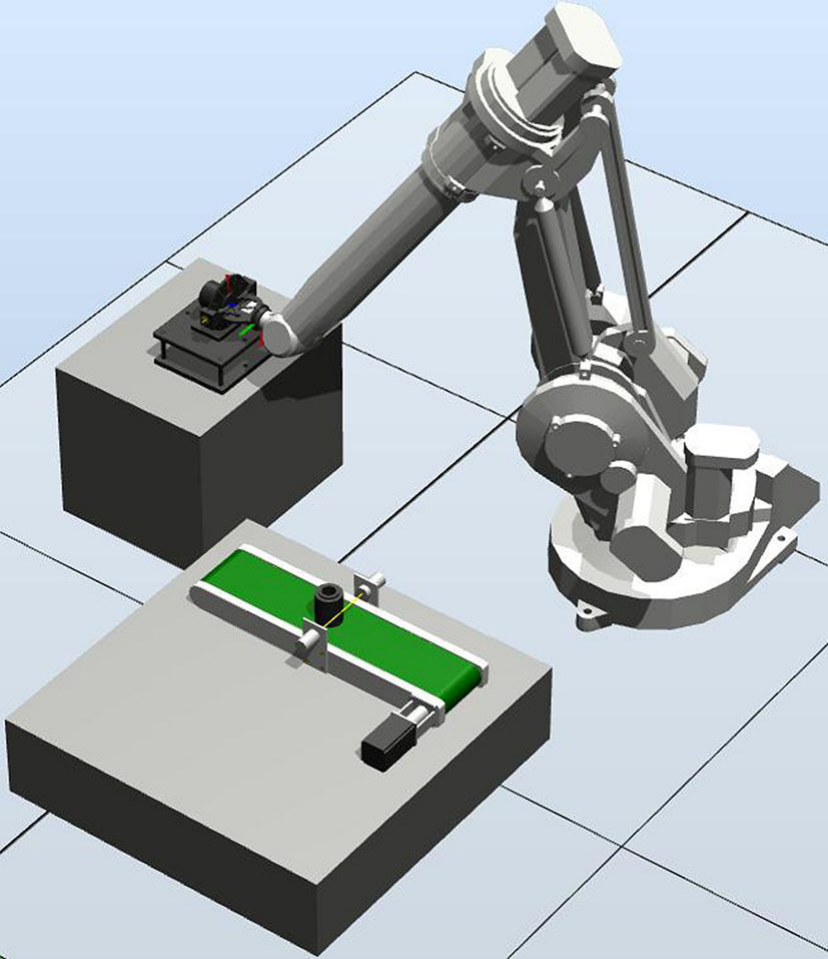
Robot Studio assembly station map.

On the basis of the previous three-dimensional work, according to the actual situation of the whole installation and transportation, this chapter uses ABB robot simulation software Robot Studio for offline programming and simulation, and applies the written and debugged program to the actual robot experiment to verify the feasibility and correctness of the whole experiment. The whole simulation includes the space design of workstations, the construction of 3D models, the design and creation of mechanical devices in software, the design and connection of smart components, the logic control between workstations, the creation of robot system and offline teaching programming. After the completion of the simulation test, the conveyor belt will move the pad parts to the corresponding position. After the completion, the robot grabs and transports the parts to the relevant position through the end effector, which can complete the whole process. In Figure 12, the robot grabs the part in the corresponding position. In Figure 13, the robot assembles the parts to the corresponding positions.

**Figure 14:**
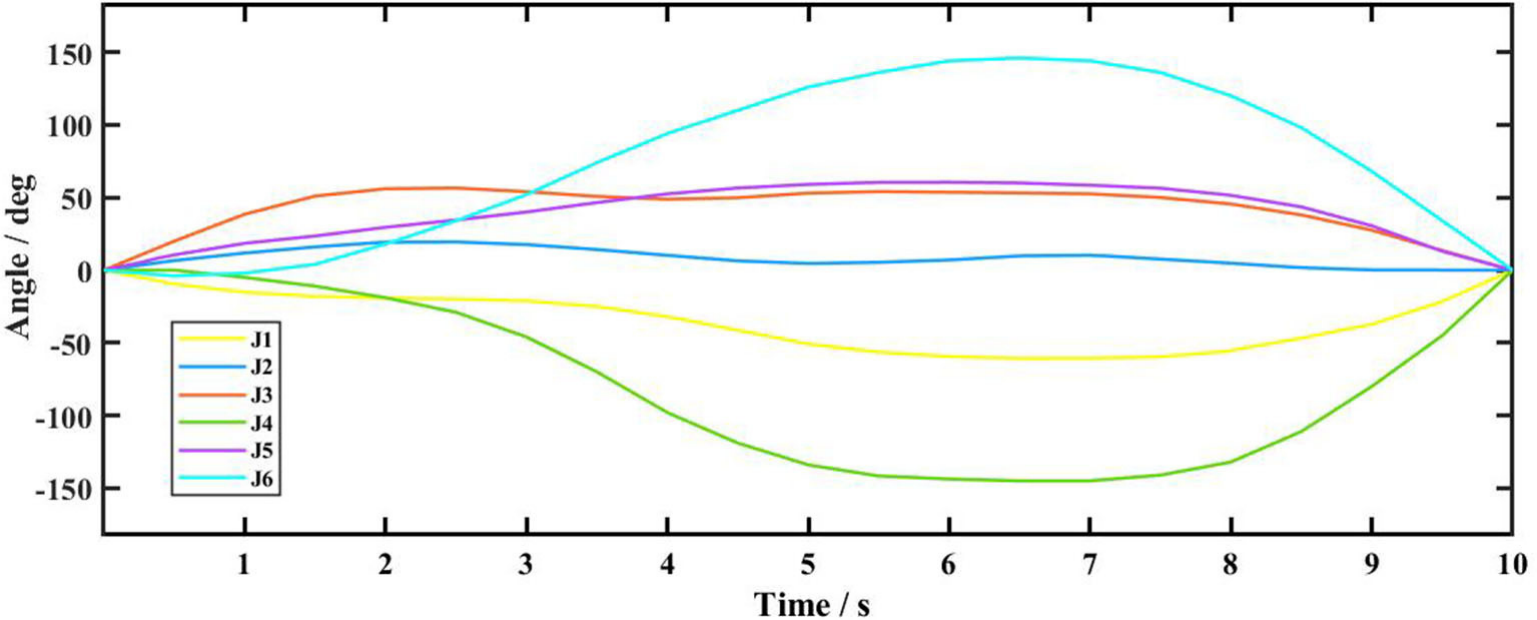
Six-axis displacement curve of robot.

After the completion of the simulation, the recorded six-axis displacement data of the robot is shown in Figure 14. In the picture, the Abscissa is the simulation time, and the ordinate is the angular displacement of six axes of the robot. J1, J2, J3, J4, J5, and J6 represent the robot's one to six axes, respectively. It can be seen in the picture that the six-axes motion of the robot move smoothly without mutation during transportation, and all of them are within the limited range, indicating that the RAPID program is correct and can be used in practical applications.

**Figure 15:**
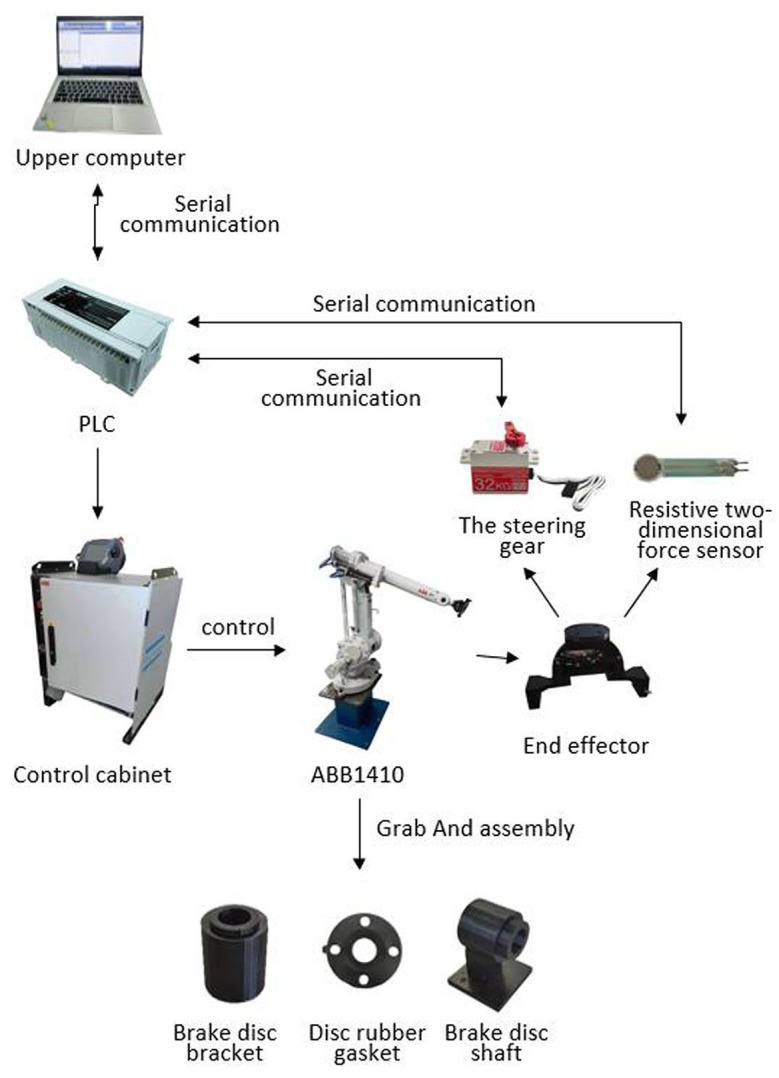
Structure diagram.

**Figure 16:**
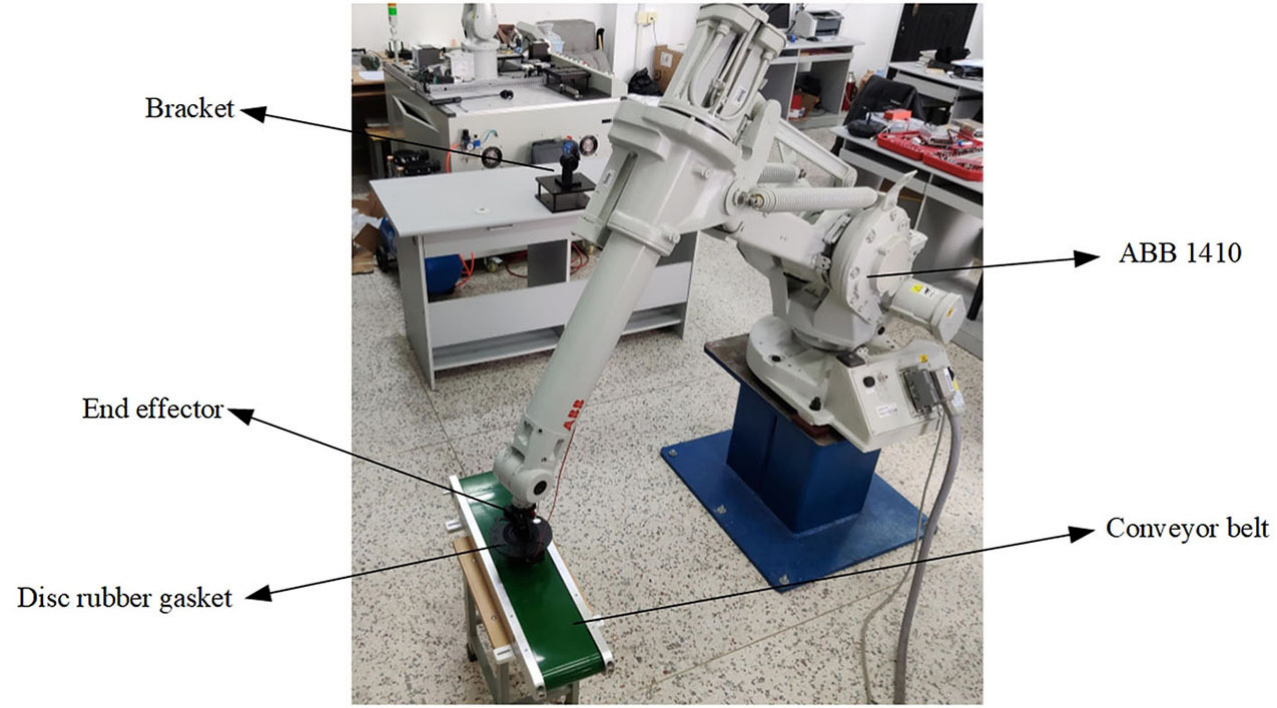
Working position diagram of experimental grasping gasket.

**Figure 17:**
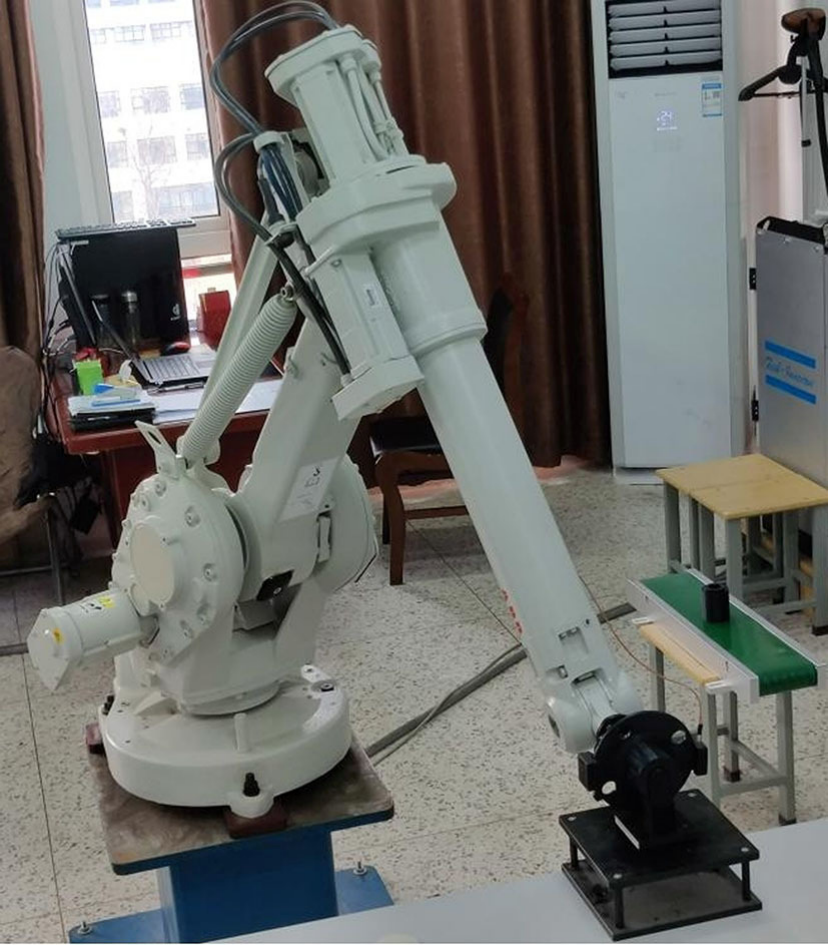
Working position diagram of experimental assembly gasket.

The composition of the whole experimental platform is shown in Figure 15. In order to show the feasibility of the experiment, the whole handling system is placed according to the preset relative space position with the robot, the program is input into the teaching device, and the experiment is carried out after adjusting the parameters. The two work maps in the experiment are shown in Figures 16, 17.

As can be seen from Figures 16, 17 that the whole experiment can achieve the stable grasping of flexible objects, and the experimental effect is consistent with that of simulation, which shows that the designed two-finger translational manipulator can achieve the initial experimental requirements of stable grasping of flexible objects when applied to the corresponding industrial robot after the introducing of two-dimensional force sensor, applied to the corresponding industrial robot can achieve the initial experimental requirements of stable grasp of flexible objects.

## Conclusion

1) A two-finger translational manipulator based on two-dimensional force sensor is designed for the small deformation and stable grasping task of disk-shaped rubber gasket. The three-dimensional model and kinematics model of the two-finger translational manipulator are established, and the corresponding relationship between the motor angle and grasp position is obtained.
2) A prototype of two-finger translational manipulator is developed, and the dynamic characteristics are studied. The experimental results show that the deformation of the disk-shaped rubber gasket increases with the increase of the grasping force, and increases obviously when the grasping force is >5 N, and the slip decreases with the increase of the grasping force. The slip decreases slowly when the grasping force is more than 5 N, and the stable grasping of small deformation can be achieved when the grasping force is 5 N.
3) Based on Robot Studio software, the handling process of the grabbing system is designed, and the verification experiment is carried out based on ABB1410. The experimental results are consistent with the simulation results, and the system can realize the small deformation and stable grasping of flexible objects.

## Data Availability Statement

The original contributions presented in the study are included in the article/supplementary material, further inquiries can be directed to the corresponding author/s.

## Author Contributions

WL: investigation, methodology, project administration, supervision, writing-review, and editing. JC: investigation, methodology, validation, and writing-original draft. PW: data curation. CJ: software. YM and KC: investigation. All authors contributed to the article and approved the submitted version.

## Conflict of Interest

The authors declare that the research was conducted in the absence of any commercial or financial relationships that could be construed as a potential conflict of interest.
